# CD3-Positive B Cells: A Storage-Dependent Phenomenon

**DOI:** 10.1371/journal.pone.0110138

**Published:** 2014-10-16

**Authors:** Angela Nagel, Christian Möbs, Hartmann Raifer, Heinz Wiendl, Michael Hertl, Rüdiger Eming

**Affiliations:** 1 Department of Dermatology and Allergology, Philipps University Marburg, Marburg, Germany; 2 Institute of Clinical and Molecular Virology, Friedrich-Alexander-Universität Erlangen-Nürnberg, Erlangen, Germany; 3 Institute for Medical Microbiology and Hygiene, Philipps University Marburg, Marburg, Germany; 4 Department of Neurology, University of Münster, Münster, Germany; University Hospital Jena, Germany

## Abstract

The majority of clinical studies requires extensive management of human specimen including e.g. overnight shipping of blood samples in order to convey the samples in a central laboratory or to simultaneously analyze large numbers of patients. Storage of blood samples for periods of time before *in vitro*/*ex vivo* testing is known to influence the antigen expression on the surface of lymphocytes. In this context, the present results show for the first time that the T cell antigen CD3 can be substantially detected on the surface of human B cells after *ex vivo* storage and that the degree of this phenomenon critically depends on temperature and duration after blood withdrawal. The appearance of CD3 on the B cell surface seems to be a result of contact-dependent antigen exchange between T and B lymphocytes and is not attributed to endogenous production by B cells. Since cellular subsets are often classified by phenotypic analyses, our results indicate that *ex vivo* cellular classification in peripheral blood might result in misleading interpretations. Therefore, in order to obtain results reflecting the *in vivo* situation, it is suggested to minimize times of *ex vivo* blood storage after isolation of PBMC. Moreover, to enable reproducibility of results between different research groups and multicenter studies, we would emphasize the necessity to specify and standardize the storage conditions, which might be the basis of particular findings.

## Introduction

Human *in vivo* studies are very difficult to realize, mostly due to ethical concerns. Thus, *ex vivo*/*in vitro* studies characterizing human immune cells and their functions are commonly applied to better understand cellular interactions and disease underlying mechanisms. In this regard, subsets of immune cells are characterized based on phenotypic markers, because surface antigens usually play a pivotal role in cell function [1]. Using dual- and multicolor flow cytometry it is very important that cells which may or may not express certain surface markers are correctly phenotyped [2]. Acquisition of different molecules by lymphocytes that are normally not transcribed by the respective cell types, may directly or indirectly influence both the phenotype and function of immune cell subsets capturing these membrane proteins and might endow the cells with features generally not associated with these cells [1], [3].

In 1993, Hultin et al. described a population of CD3^+^ T cells expressing low amounts of the B cell antigen CD20 on their cell surface [4]. Recent reports confirmed this finding and postulated a functional importance of these cells, since CD20^+^ T cells are found to represent a terminally differentiated cell type with immunoregulatory and proinflammatory capacity [5], [6]. With the exception of CD20, these T cells did not express any other B cell marker and treatment of patients suffering from rheumatoid arthritis (RA) with rituximab led to depletion of both peripheral CD20^+^ B cells and CD20^+^ T cells [5], [6]. Rituximab is a chimeric monoclonal antibody directed toward CD20 that has proven very effective in depleting normal and malignant B lymphocytes and is widely used in the treatment of B cell malignancies and several autoantibody-mediated autoimmune diseases such as RA, systemic lupus erythematosus, primary Sjögren’s syndrome, idiopathic thrombocytopenic purpura and pemphigus vulgaris (PV) [7]–[14].

Since we were interested in the impact of rituximab on B cell depletion [14], [15], we enlarged our studies on the presence of the aforementioned CD20^+^ T cells within the peripheral blood mononuclear cells (PBMC) fraction of PV patients. Interestingly, we could identify a population of CD3-expressing CD20^+^ B cells (CD3^low^CD20^+^ B cells) in PBMC of PV patients. More detailed analyses investigating peripheral blood of additional patient cohorts suffering from autoimmune or allergic diseases and healthy controls demonstrated that the appearance of CD3^low^CD20^+^ B cells was a disease-unrelated phenomenon resulting from overnight (oN) storage of blood or PBMC samples at non-physiological low temperatures. Furthermore, our results show that CD3 is not endogenously produced by B cells, as described for CD20 expression in the case of T cells [6].

The observed phenomenon of CD3 appearance on B cell surfaces might challenge the current view that oN or long-term storage of peripheral human blood samples – a prerequisite in many clinical trials – are appropriate procedures reliably preserving the *in vivo* situation of immunological processes and cellular characteristics.

## Materials and Methods

### Patients

Blood samples were obtained from a total of 62 adult donors consisting of 32 patients with chronic inflammatory skin diseases (17 PV patients, 2 pemphigus foliaceus patients, 6 patients with psoriasis, 4 patients with bullous pemphigoid, 2 patients with systemic lupus erythematosus, 1 patient with epidermolysis bullosa acquisita), 13 patients with immediate-type allergies, and 17 healthy controls. All patients were recruited from the Department of Dermatology and Allergology, Marburg, Germany, following written informed consent. The study was approved by the Ethics Committee of the Medical Faculty of Marburg and it was conducted according to the Declaration of Helsinki Principles.

### Blood samples and isolation of peripheral blood lymphocytes

Citrate-phosphate-dextrose-adenine (CPDA) containing blood samples were taken from patients and healthy controls. To exclude an impact of the applied anticoagulant both CPDA and ethylene-diamine-tetra-acetate (EDTA) containing blood samples were analyzed. Blood samples were either processed within 3 hours (freshly isolated PBMC) or stored before isolation of lymphocytes oN at room temperature (RT) and at 4°C, respectively. PBMC were isolated from blood samples by Pancoll (PAN-Biotech, Aidenbach, Germany) density gradient centrifugation for cell culture experiments and magnetic cell separation (MACS), or blood samples were processed by ACK lysis (lysing buffer: 150 mM NH_4_Cl, 1 mM KHCO_3_ and 0.1 mM EDTA) for flow cytometry.

In one set of experiments blood samples were additionally treated with different amounts of the monensin-containing protein transport inhibitor BD GolgiStop (BD Biosciences, Heidelberg, Germany) for the 12 h of oN at 4°C (oN/4°C) storage.

### Magnetic cell separation

CD20^+^ B lymphocytes (positive selection) and CD4^+^ T lymphocytes (negative selection) were purified according to their respective surface markers using MACS following the manufacturer’s protocol (Miltenyi Biotec, Bergisch Gladbach, Germany). Purities of CD20^+^ and CD4^+^ cells were routinely 93–96% of lymphocytes as calculated by flow cytometry. Finally, MACS-isolated CD20^+^ and CD4^+^ cells were quantified with a hemocytometer and subjected to polymerase chain reaction (PCR) or co-culture experiments.

### Cell culture

For time kinetic studies isolated PBMC were cultured at a concentration of 2×10^6^ cells/ml in RPMI 1640 medium supplemented with 100 U/ml penicillin, 100 µg/ml streptomycin and 2 mM L-glutamine (all from PAA Laboratories, Cölbe, Germany) for 24–96 h at RT, in a humidified atmosphere containing 5% CO_2_ at 37°C, or in a refrigerator at 4°C before flow cytometric analysis.

### Co-culture and transwell experiments

Co-culture experiments were performed using MACS-isolated CD4^+^ T cells and CD20^+^ B cells at ratios of 1∶1, 4∶1 and 9∶1, respectively, at a final concentration of 2×10^6^ cells/ml. In the case of antigen fixation, CD4^+^ T cells were incubated with 1% paraformaldehyde (PFA) for 10 min before co-culture with CD20^+^ B cells. In transwell experiments, a total of 1.6×10^6^ or 1.8×10^6^ CD4^+^ T cells were placed with 0.4×10^6^ or 0.2×10^6^ CD20^+^ B cells (i.e. ratios of 4∶1 and 9∶1) in transwell chambers (Thincert, 0.4 µm, Greiner Bio One, Frickenhausen, Germany). Both B and T cells were analyzed after 24 h of either co-culture or transwell experiments by flow cytometry as described below.

### Flow cytometry

The expression of distinct surface molecules on lymphocytes was determined by flow cytometry. Cells were simultaneously immunostained using the following anti-human antibodies: CD3-APC (UCHT1), CD4-APC (RPA-T4), CD5-PE (UCHT2), CD19-PE (HIB19), CD20-FITC (2H7), CD27-PE (M-T271), CD80-PE (L307.4), CD86-PE (2331/FUN-1), IgG-PE (G18-145), αβ-TCR-PE (T10B9.1A-31), γδ-TCR-PE (B1), BAFF-R-FITC (11C1), or respective isotype controls (all from BD Biosciences, Heidelberg, Germany). In brief, cells were stained for 30 min on ice in the dark. After two additional washing steps, 3×10^5^ cells (or 1×10^5^ cells for co-culture/transwell experiments, respectively) were analyzed by flow cytometry (FACS Calibur, BD Biosciences, Heidelberg, Germany) using CellQuest and WinMDI software. The following gating strategy was performed: lymphocytes were identified by forward and side scatter characteristics. Subsequently, applying appropriate isotype controls, the CD20^+^ cell population was defined and divided into CD3^+^ (CD3^low^) and CD3^-^ cells, which were utilized for further phenotyping analysis. Cell viability was evaluated by 7-aminoactinomycin D (7-AAD) staining (Via-Probe, BD Biosciences, Heidelberg, Germany).

In a subset of experiments, sorting of CD3^low^C20^+^ cells was performed using a FACS Aria III (BD Biosciences, Heidelberg, Germany) and cells were analyzed by the FlowJo software (Tree Star Inc., Ashland, USA).

### Immunofluorescence microscopy

Confocal images of living B cells were acquired on a Leica TCS SP2 AOBS laser scanning microscope using a 40× oil Plan-Apochromat lens (LSM, Leica Microsystems, Wetzlar, Germany). CD20^+^ cells were purified from whole blood samples after oN storage at 4°C, i.e. PBMC were isolated by density gradient centrifugation followed by MACS of CD20^+^ cells. Afterwards 5×10^4^ CD20^+^ cells were immunostained with CD20-FITC (2H7), CD19-PE (HIB19), CD3-PE (UCHT1) antibodies or the respective mouse IgG1,κ and IgG2b,κ isotype controls (all BD Biosciences Heidelberg, Germany). Data evaluation and co-localization analysis was performed with region of interest (ROI) detection of the LEICA software in combination with the IMARIS imaging software package.

### RNA isolation and PCR amplification

Total RNA was extracted from 7×10^5^ MACS-purified B cells and a T cell line (TCL) as positive control using the RNAeasy kit (Qiagen, Hilden, Germany). cDNA was synthesized using the Omniscript RT kit (Qiagen, Hilden, Germany), oligo(dT) primers, random primers and RNasin (all from Promega, Mannheim, Germany). PCR amplification was performed with 100 ng cDNA (TCL: 50 ng cDNA) on a Hybaid thermal PCR cycler (Thermo Electron, Langenselbold, Germany) using dNTP Mix, DNA Taq Polymerase (both from Peqlab, Erlangen, Germany) and specific oligonucleotide primers (Thermo Electron, Ulm, Germany). The CD3-specific sense primer sequence was: 5′-AAGATGGTTCGGTACTTCTGACTTGTG-3′; the antisense primer was: 5′-GTAGAGCTGGTCATTGGGCAACAGAGT-3′. The β-actin specific sense primer was: 5′-CTAGAAGCATTTGCGGTGGACGATGGAGGG-3′; the antisense primer was: 5′-TGACGGGGTCACCCACACTGTGCCCATCTA −3′. The PCR conditions were as follows: 35 cycles of 1 min 94°C, 1 min 60°C and 2 min 74°C as described by Fayette et al. [16].

### Statistical analysis

Considering the non-normal distribution of the generated data, non-parametric tests were used and continuous variables are shown as median with quartiles and range, illustrated as box-whisker plots. The boxes contain 50% of the data (25^th^ and 75^th^ percentiles), whereas median values are represented by center lines. The whiskers restrict the minimum and the maximum of the data set. Outliers (distance from 1st or 3rd quartile >1.5× length of the box) are illustrated as circles and extreme values (distance from 1st and 3rd quartile >3× length of the box) as asterisks, respectively. For comparison of two independent groups of donors classified according to analysis of freshly isolated PBMC and PBMC isolated after storage of blood samples oN/4°C, the two-sided Mann-Whitney-U-Test was used. Level of significance α was defined as less than 0.05 (p<0.05). For comparison of different patient groups classified according to their underlying disease the Kruskal-Wallis-Test was applied. In the case of p<0.05, differences between two groups were compared. Apart from that, results of n≤4 independent experiments are shown as mean ± SEM. Statistical analysis was performed using SPSS 12.0.

## Results

### Detection of T cell antigens on the cell surface of CD20^+^ cells under non-physiological conditions

**Figure 1:**
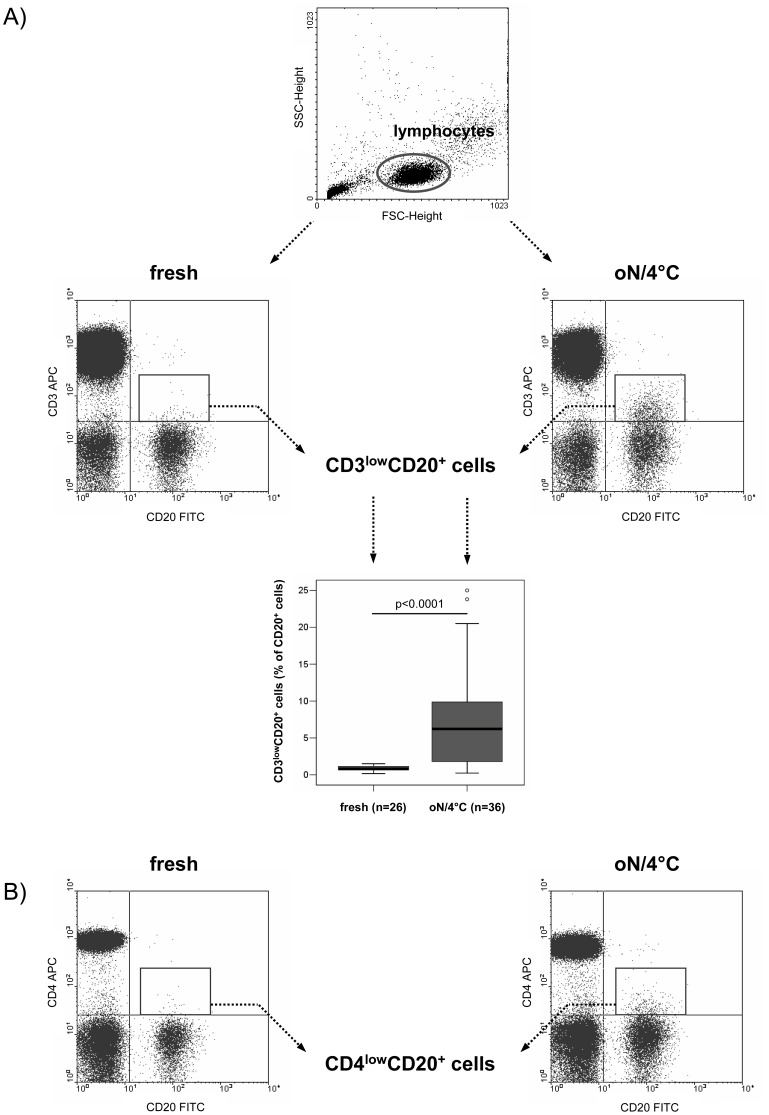
Detection of the T cell antigens CD3 and CD4 on CD20^+^ lymphocytes by flow cytometry. The appearance of both CD3 (A) and CD4 antigens (B) on the surface of CD20^+^ lymphocytes is shown for one representative patient. Freshly isolated lymphocytes (left) were compared to lymphocytes analyzed after overnight (oN) storage of whole blood samples at 4°C (right). The statistically significant difference in the number of CD3-expressing CD20^+^ (CD3^low^CD20^+^) lymphocytes between two independent groups of donors (fresh *versus* oN/4°C) was determined by the two-sided Mann-Whitney-U-Test. Outliers are depicted as circles.

Immunostaining for CD3 and CD20 on the cell surface of freshly isolated lymphocytes compared to lymphocytes isolated after oN/4°C storing of blood samples revealed a distinct population of low CD3 expressing CD20^+^ cells (CD3^low^CD20^+^ cells) upon oN/4°C storage. Figure 1A illustrates the results of a representative patient, whose freshly isolated lymphocytes contain a comparably small population of CD3^low^CD20^+^ cells (1.76% of CD20^+^ cells), whereas this population is strongly increased after oN/4°C storage (16.29% of CD20^+^ cells). Apparently, similar findings were seen with other T cell antigens such as the CD4 co-receptor (0.91% *versus* 4.46% CD20^+^ cells; Figure 1B) as illustrated for the same patient. Comparative statistical analysis of PBMC isolated from fresh (n = 26) *versus* oN/4°C (n = 36) blood samples showed significantly increased numbers of CD3^low^CD20^+^ cells after oN/4°C storage as assessed by flow cytometry (p<0.0001; Figure 1A). However, analysis of patients regarding the underlying disorders (cf. Materials and Methods) did not show any disease-related differences in numbers of CD3^low^CD20^+^ cells (p>0.05, data not shown). Moreover, CD3^low^CD20^+^ cells were detectable after oN/4°C incubation, irrespective of using CPDA- or EDTA-containing blood samples, excluding an anticoagulant-related effect (data not shown). In addition, CD3^low^CD20^+^ cells were found after oN/4°C storage of both whole blood samples and isolated PBMC (stored in cell culture medium).

### Detection of CD3 on CD20^+^ cells by flow cytometry is not a staining artifact

**Figure 2:**
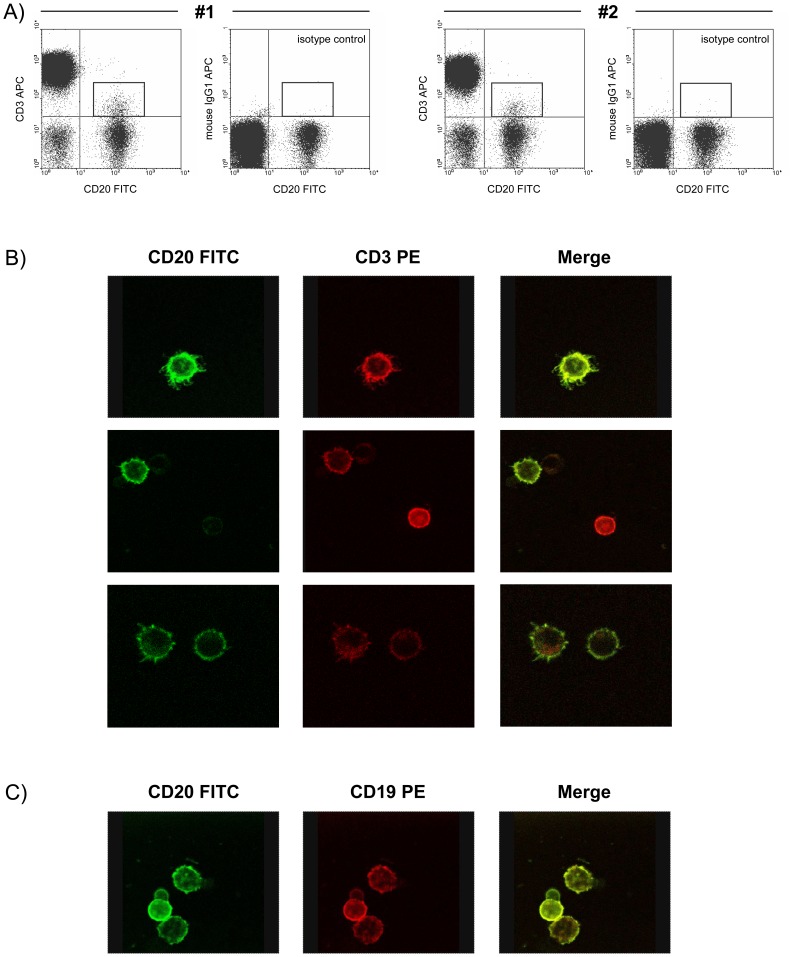
Co-expression of CD3 and CD20 on the cell surface is not a staining artifact. (A) Shown is the presence of CD3^low^CD20^+^ cells after overnight storage of blood samples at 4°C for two representative patients (#1 and #2, left). Staining of blood cells with the respective allophycocyanin (APC)-labeled mouse IgG1 isotype control excludes an artifact caused by the fluorochrome APC (#1 and #2, right). (B) Simultaneous expression of CD20 and CD3 on the surface of MACS-purified CD20^+^ lymphocytes is demonstrated for one representative donor by single cell analysis using confocal laser microscopy. (C) Confirmation of concomitant CD20 (FITC) and CD19 (PE) expression on MACS-purified CD20^+^ B cells served as positive control. Whole blood samples were stored overnight at 4°C before PBMC isolation and MACS analysis.

Since it is known that allophycocyanin (APC) is a large fluorescent protein, which could bind non-specifically to the cell surface of lymphocytes, and that double-staining artifacts were observed in certain individuals during dual color immunophenotyping [2], experiments were performed using both APC-labeled anti-human CD3 (UCHT1) and mouse IgG1,κ (isotype control) antibodies. Figure 2A demonstrates in two representative patients (#1 and #2) that CD3 was detectable on the surface of CD20^+^ cells after oN/4°C storing of blood samples (Figure 2A, left) compared to APC-labeled isotype control (Figure 2A, right) excluding a non-specific binding of APC, e.g. to Fc receptors on the surface of lymphocytes.

To verify the observation of CD3 expression on CD20^+^ cells after oN/4°C storage, single cell analysis of MACS-purified CD20^+^ cells (after oN/4°C storage of whole blood) was performed by confocal laser microscopy utilizing a PE-labeled anti-human CD3 antibody. Figure 2B demonstrates the detection of both FITC-labeled CD20 (Figure 2B, left) and PE-labeled CD3 (Figure 2B, center) on the surface of single cells of a representative patient. Merging of the images proved the simultaneous expression of both antigens on the cell surface of MACS-purified CD20^+^ cells (Figure 2B, right). Flow cytometric analysis of MACS-purified CD20^+^ cells of the same donor using APC-labeled CD3 and FITC-labeled CD20 antibodies (analogous to Figure 1A) revealed a proportion of 11.11% CD3^low^CD20^+^ cells on CD20^+^ cells (data not shown). The confirmation of concomitant CD20 (FITC) and CD19 (PE) expression on MACS-purified CD20^+^ B cells served as a positive control (Figure 2C).

Since an identical CD3 antibody clone (UCHT1) was used for both flow cytometric analysis (APC-labeled) and confocal laser microscopy (PE-labeled), a potential clone-specific staining artifact might be taken into account. However, using an APC-labeled anti-human CD4 antibody (clone RPA-T4), CD4^low^CD20^+^ cells were also be detected by flow cytometric analysis (Figure 1B) excluding an antibody-specific staining artifact.

### CD3^low^CD20^+^ cells belong to the B cell population

**Figure 3:**
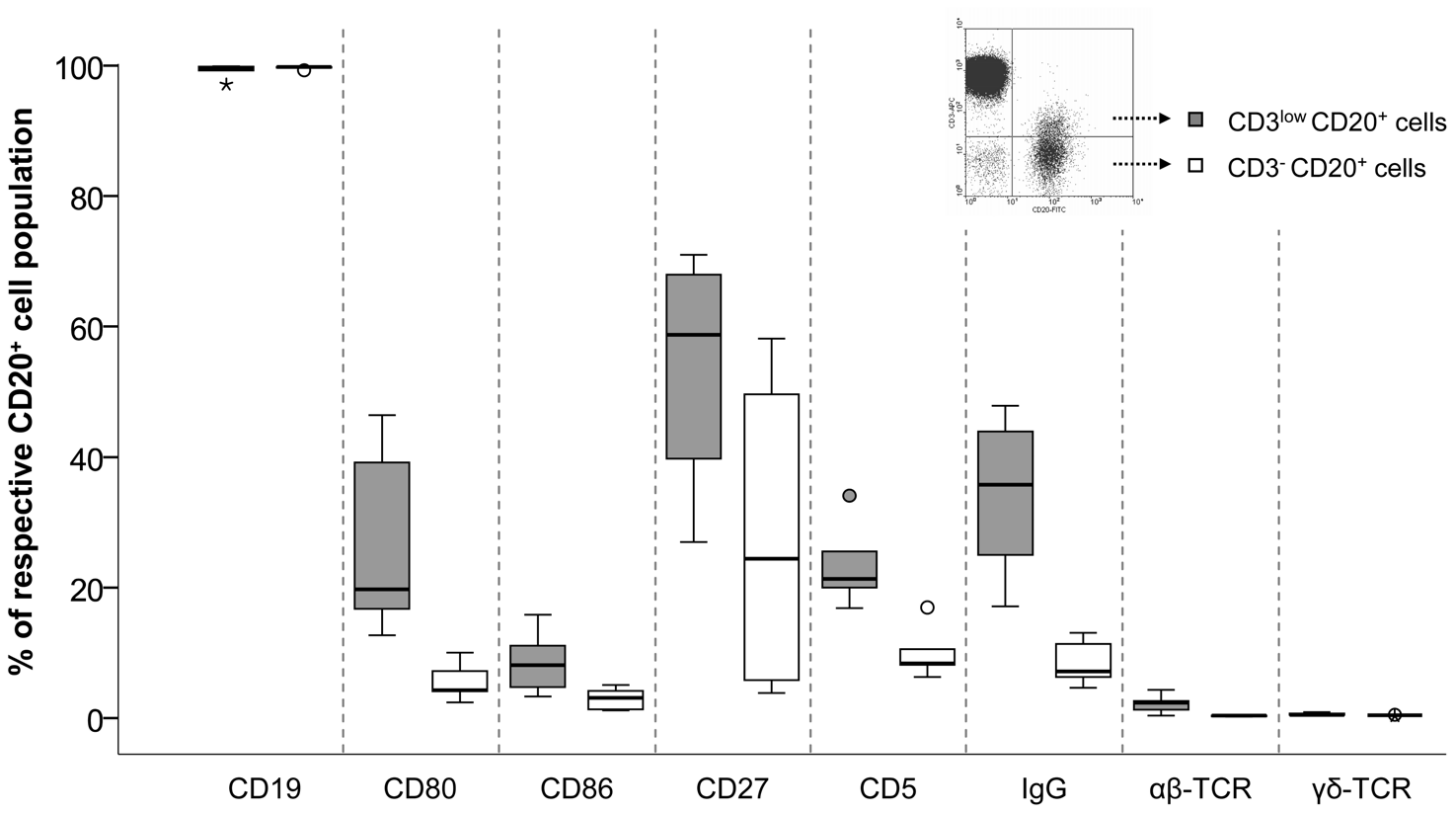
CD3^low^CD20^+^ cells belong to the B lymphocyte compartment. Phenotyping of CD3^low^CD20^+^ cells revealed CD19 expression in all of the cells, confirming the assignment of CD3^low^CD20^+^ cells to the population of B lymphocytes. B cells showing characteristic features of an activated (CD80, CD86, CD5) and memory (IgG) phenotype are elevated in CD3^low^CD20^+^ B cells compared to the CD3^-^CD20^+^ B cell subset. Additionally, expression of the specific T cell markers αβ- and γδ-TCR were only marginally found on the cell surface of CD3^low^CD20^+^ and CD3^-^CD20^+^ B cells, respectively, excluding that the CD3^low^CD20^+^ cells are T-B-cell doublets. Extreme values are illustrated as asterisks, outliers as circles. Shown are the results from at least 5 individual patients. Comparative statistical evaluation of different B cell subset regarding cell surface antigen expression was not performed due to the small sample size.

Phenotypic characterization by means of flow cytometry revealed that both CD3^low^CD20^+^ and CD3^-^CD20^+^ cells belong to the population of B lymphocytes (Figure 3). The cells of both subsets were found to express CD19 (Figure 3) and BAFF-R (>98%; data not shown) on their cell surface. Furthermore, both barely expression of specific T cell markers such as αβ-TCR and γδ-TCR (Figure 3) and doublet discrimination by flow cytometry (Figure S1) confirmed that the CD3^low^CD20^+^ lymphocyte population did not consist of T-B-cell clusters. Compared to CD3^-^CD20^+^ B cells the population of CD3^low^CD20^+^ B cells contained a substantially higher amount of CD5^+^, CD80^+^, CD86^+^ and surface IgG^+^ cells (Figure 3). Thus, the CD3^low^CD20^+^ subset predominantly encompasses B cells with an activated or memory phenotype.

### CD3 is not endogenously produced by B lymphocytes

**Figure 4:**
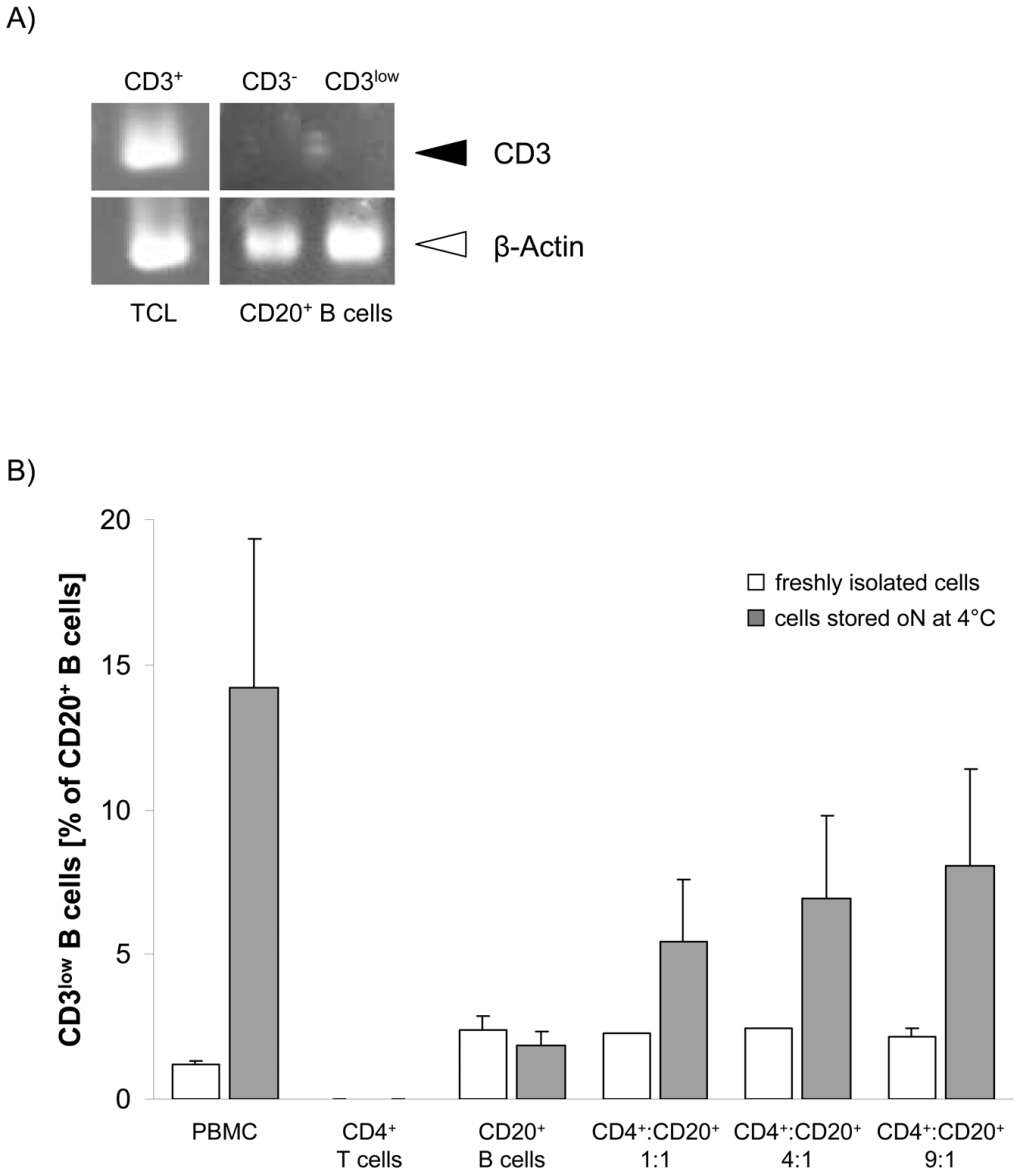
Detection of CD3 on B cell surfaces is a result of cell number- dependent T-B cell interactions. Semiquantitative RT-PCR for CD3 was performed using RNA isolated from 7×10^5^ MACS-purified CD20^+^ B cells and a CD3-expressing T cell line (TCL). (A) Endogenous expression of CD3 was only found in the TCL, which served as a positive control, but neither in CD3^-^CD20^+^ nor in CD3^low^CD20^+^ B cells. β-actin was used as loading control. (B) The appearance of CD3^low^CD20^+^ B cells was demonstrated after co-culture of MACS-purified CD4^+^ T cells and CD20^+^ B cells in different ratios overnight (oN) at 4°C. Storage of CD20^+^ B cells alone under the same conditions was not associated with an induction of the CD3^low^CD20^+^ B cell population, pointing at the need for T cells. Furthermore, the quantity of CD3^low^CD20^+^ B cells was dependent on the number of CD4^+^ T cells, i.e. increasing T-B cell ratios caused elevated numbers of CD3^low^CD20^+^ B cells. Shown are the results of up to three independent experiments.

Endogenous expression of CD3 mRNA in CD3^low^CD20^+^ B cells was examined using RT-PCR. As shown in Figure 4A, CD3 mRNA was neither detected in the MACS-purified CD3^-^CD20^+^ B cell subset nor in the MACS purified CD20^+^ B cells containing about 20% CD3^low^CD20^+^ cells, as determined by flow cytometry (data not shown). In contrast, amplified CD3 cDNA was detected using a CD3^+^ TCL as a positive control (Figure 4A).

This finding was confirmed by experiments applying freshly MACS-isolated CD4^+^ T cells and CD20^+^ B cells. Only oN/4°C storage of CD20^+^ B cells alone did not induce any increase in CD3^low^CD20^+^ B cell numbers compared to freshly isolated CD20^+^ B cells (Figure 4B). In contrast, oN/4°C co-culture of isolated CD4^+^ T cells with equal numbers of CD20^+^ B cells resulted in markedly elevated numbers of CD3^low^CD20^+^ B cells. This T cell-dependent increase of CD3 antigen on the B cell surface was directly related to the T-B cell ratio, i.e. increasing numbers of CD4^+^ T cells caused elevating numbers of CD3^low^CD20^+^ B cells within the co-cultures (Figure 4B).

### Transfer of CD3 from T to B cells is cell contact-dependent and impaired by monensin treatment

**Figure 5:**
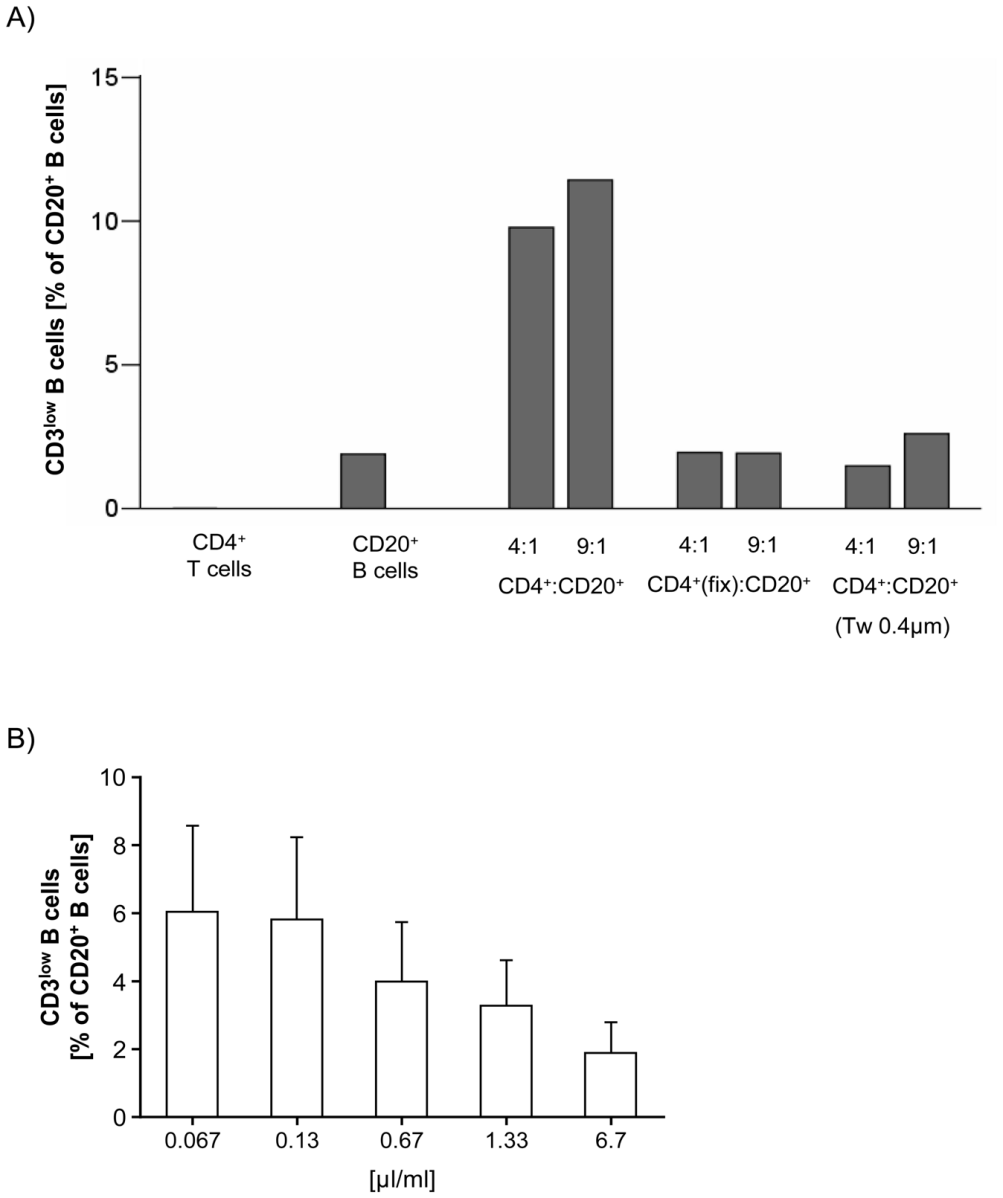
Transfer of CD3 from T to B cells requires cell-cell contact and is impaired by monensin treatment. (A) Shown are numbers of CD3^low^CD20^+^ B cells in one patient after co-culture of MACS-purified CD4^+^ T cells and CD20^+^ B cells overnight (oN) at 4°C. While CD3^low^CD20^+^ B cells were found to be elevated with increasing T-B cell ratios, fixation of antigens on the T cell surface using 1% PFA before co-culture with B cells oN at 4°C did prevent CD3 acquisition by CD20^+^ B cells. Moreover, co-culture of CD4^+^ T cells and CD20^+^ B cells using transwell (Tw) chambers (pore size of 0.4 µm) prevented the appearance of CD3^low^CD20^+^ B cells, excluding the possibility that membrane vesicles like exosomes transfer T cell surface markers from T to B cells. (B) The occurrence of CD3^low^CD20^+^ B cells after oN storage of blood samples at 4°C was impaired by the addition of monensin (applied by the protein transport inhibitor BD GolgiStop). Shown are the results of four independent experiments.

The pronounced increase of CD3^low^CD20^+^ B cell numbers was confirmed after oN/4°C co-culture of both MACS-purified fresh CD4^+^ T cells and CD20^+^ B cells (ratio 4∶1 *versus* 9∶1) compared to CD20^+^ B cells alone (Figure 5A). In contrast, same conditions but fixation of antigens on the T cell surface using 1% PFA before co-culture with CD20^+^ B cells prevented the occurrence of CD3^low^CD20^+^ B cells (Figure 5A). Additionally, separation of CD4^+^ T cells and CD20^+^ B cells using transwell chambers did not result in elevated CD3^low^CD20^+^ B cell numbers compared to CD20^+^ B cells alone (Figure 5A). Therefore, transfer of CD3 from T cells to B cells might not depend on small membrane vesicles such as frequently described exosomes, since these nanovesicles – with a diameter of 30–100 nm [17], [18] – are able to pass the transwell membrane (pore size: 400 nm) [19], [20].

Treatment of whole blood samples with increasing amounts of the monensin-containing protein transport inhibitor BD GolgiStop during oN/4°C storage led to decreased numbers of CD3^low^CD20^+^ B cells (Figure 5B). Since it is known that the carboxylic ionophore monensin is involved in distinct physiologic processes, like perturbation of the golgi apparatus and inhibition of both vesicle trafficking by lysosomes and molecule recycling from early endosomes, decreased numbers of CD3^low^CD20^+^ B cells indicate that intracellular protein transport mechanisms are involved in this phenomenon.

### Numbers of CD3^low^CD20^+^ B cells depend on duration and temperature of storage

**Figure 6:**
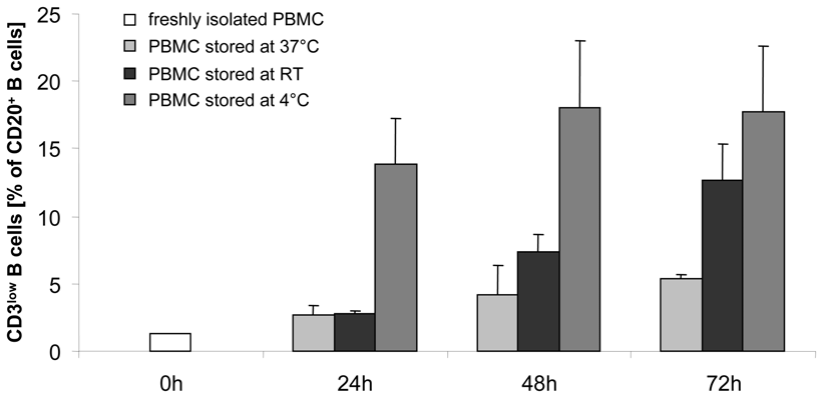
Numbers of CD3^low^CD20^+^ B cells are time- and temperature-dependent. The increase in the number of CD3^low^CD20^+^ B cells was time-dependent, but independent of storage conditions (4°C *versus* room temperature (RT) *versus* humidified atmosphere at 37°C, 5% CO_2_). CD3^low^CD20^+^ B cells were detectable at earlier time points and more pronounced at 4°C storage compared to RT and 37°C incubation, respectively. Shown are the results of two independent experiments performed in the same patient.

Following B cells over time of storage revealed increasing numbers of CD3^low^CD20^+^ cells, independent of the storing condition (humidified atmosphere at 37°C and 5% CO_2_ *versus* RT *versus* 4°C) compared to freshly isolated cells (Figure 6). However, the largest increase in CD3^low^CD20^+^ cells was observed after storage of isolated PBMC at 4°C. Moreover, each of the tested storing conditions resulted in a time-dependent increase in CD3^low^CD20^+^ B cells compared to freshly isolated cells (Figure 6). Thus, it seems that non-physiological storing conditions (4°C>RT >37°C) of blood samples might favor the generation of CD3^low^CD20^+^ B cells.

## Discussion

In the present study, we show for the first time that T cell antigen CD3 can be detected on the surface of B cells due to storage of human lymphocytes under critical low temperatures (oN/4°C). Moreover, *ex vivo* storage of whole blood samples/PBMC at either RT or in a humidified atmosphere (37°C, 5% CO_2_) for more than 24 h likewise induced the population of CD3^low^CD20^+^ B cells. Increases in numbers of these cells were noticed in a time- and temperature-dependent manner. Furthermore, CD3 was not endogenously produced by B lymphocytes, but is a result of cell contact-dependent transfer from T to B cells. Thus, our results provide new insights into the limitations of *ex vivo* studies analyzing human lymphocyte subsets.

The present results are in line with recent reports demonstrating that storage of blood samples for extended periods of time before analysis influences the phenotyping of lymphocytes and that the degree of this *ex vivo* phenomenon critically depends on the temperature [21]–[23]. It has been described that blood samples stored at 4°C show less immunomodulatory changes than blood kept at RT [23]–[26] and that a temperature of around 4°C is the optimum storage condition for blood samples, if B lymphocytes need to be tested [22]. Therefore, it is recommended to store blood samples at 4°C/oN to reduce the metabolism of cells and prevent B cell loss, if an immediate analysis of blood cannot be realized. In contrast, our results show that, compared with freshly analyzed blood cells, a strong detection of CD3 antigen was noted on B cells already 24 h after 4°C storage of blood samples. Since immune cells are often phenotypically classified only by surface molecules using dual- and multicolor flow cytometry, it is of particular importance to avoid processes of *ex vivo* cell surface protein modification [1], [2] in order to minimize the risk for misinterpretation of storage-induced changes as clinical or pathological findings.

A major finding of this study is the increase in CD3^low^ B cell numbers over time and by reducing the storage temperature, presumably as a result of non-physiological storing conditions. Therefore, to prevent misleading interpretations, e.g. regarding a potential induction or suppression of T cells during a course of disease or treatment, it is important to apply constant storage conditions during a study. Furthermore, storing conditions of blood samples have to be explicitly described in the study design of published manuscripts to ensure reproducibility of results, to minimize interlaboratory variation and to enable appropriate conclusions.

However, the detection of CD3^low^CD20^+^ cells was independent of any underlying disease, since the induction of CD3^low^CD20^+^ B cells was comparable in patients with different skin or allergic diseases and healthy controls, respectively. To exclude an effect of CPDA, different anticoagulants, such as EDTA, were used for blood collection with comparable results regarding the numbers of CD3^low^CD20^+^ cells. In addition, the possibility that the appearance of CD3 on B cells was merely an artifact caused by fluorescent co-expressing staining [2] or presence of T-B cell doublets [27] could be excluded.

Since endogenous expression of CD3 by B cells can be ruled out and appearance of CD3 on B cell surface is strongly dependent on T cell contact, it is reasonable to assume that CD3 expression on B cells is a result of antigen acquisition from T cells. Intercellular contacts which could account for the process of cell protein exchange include trogocytosis [1], [19], [28]–[37], long membrane nanotubes [38], [39], membrane bridges [38], and membrane vesicles like exosomes [17], [18], [38], [40]–[42]. Trogocytosis is a well-documented mechanism of antigen exchange between interacting cells [43] which is observed in T and B lymphocytes, natural killer cells, antigen presenting cells, monocytes and tumor cells [1], [28], [44]–[48] and is characterized by a fast and transient, unidirectional, cell contact-dependent selective protein transfer between adjacent cells [19], [29]–[31], [33], [37], [39]. There are several findings supporting the hypothesis that the detection of CD3 antigen on B cells may be a result of trogocytosis [1], [29], [37], [49]. B cells unlike T cells were reported to adopt surface molecules even at 4°C reflecting a signaling-independent passive process of antigen exchange [1], [19], [30] supporting the data of CD3 expression on B cells stored at 4°C. Furthermore, antigen non-specific trogocytosis is a feature of fully activated and differentiated effector cells [34], [36], [37], [44]. Our results show that compared to normal CD3^-^CD20^+^ B cells, there is a higher number of cells expressing activation markers such as IgG and co-stimulatory molecules like CD80 on the cell surface in the CD3^low^CD20^+^ B cell compartment. In this regard, B cells carrying CD5, which is described to be transiently induced on activated human B cells [50]–[52], were also increasingly found in CD3^low^CD20^+^ B cells. Additionally, uptake of CD3 by B cells is strictly dependent on cellular contact, since separating T and B cells by semipermeable transwell membranes completely inhibited the detection of CD3^low^CD20^+^ cells. Fixation of T lymphocytes with 1% PFA prior to co-incubation with B cells strongly inhibits CD3 acquisition by B cells as well. Apart from that, it was recently shown that CD3 is present in exosome-like microvesicles released by human T cells [42], [53]. Exosomes are secreted by several cell types such as dendritic cells, B and T lymphocytes, platelets, epithelial cells, tumor cells as well as mast cells [1], [17]–[19], [40], [41]. These small 30–100 nm diameter membrane vesicles are not involved in the herein described uptake of the T cell antigen CD3 by B cells, because separation of T and B lymphocytes using a transwell membrane with a pore size of 400 nm (enabling exosome transfer from T to B cells) strongly inhibited antigen exchange.

Several studies described a small population of CD20 expressing CD3^+^ T cells in patients suffering from RA [5], [6]. Given that cell surface molecules expressed on leukocytes have functional relevance for these cells, it is possible that CD20 plays a role in the function of those T cells [4]. In this regard, CD20^+^ T cells represent a highly activated cell population co-expressing several activation markers and producing cytokines constitutively [6]. In T cells the CD3 complex is crucial in transducing antigen recognition signals and plays a role in TCR-induced growth arrest, cell survival and proliferation [54], [55]. To our knowledge, the functional relevance of CD3 on B cells, which is not endogenously produced by B cells but rather appears to be passively acquired from T cells, remains unknown. Sabzevari et al. described that acquisition of CD80 from antigen-presenting cells by activated CD4^+^ T cells leads to capability of antigen presentation [56]. Therefore, cell contact-dependent acquisition of CD3 by B cells might be an answer to detrimental conditions promoting the survival of respective B cells through inhibition of apoptosis or leading to improvement of outside-in signaling via not yet known cellular pathways and mechanisms.

This report focuses on the description and phenotypic characterization of CD3^low^ B cells to give new insights into the degree of storage-induced cellular alterations which should be considered when performing human *ex vivo*/*in vitro* studies. Nonetheless, CD3 expression on B cells seems to be not only a storage-induced phenomenon. Four cases of large B cell lymphoma aberrantly co-expressing the T cell marker CD3 on B cells in tissue specimens were described by Wang et al., indicating that use of CD3 antibodies alone may lead to an incorrect classification of cell lineage in some B cell non-Hodgkin lymphomas [57]. Additionally, Rizzo et al. reported a case of T cell lymphoma showing aberrant co-expression of CD19 on CD3^+^ T cells in lymph node specimens as well as peripheral blood [58] and recent investigations found CD20-expressing CD3^+^ T cells in RA patients [5], [6]. In this context, there might be a putative biological relevance of the CD3 receptor on B cells. Nevertheless, further characterization of this B cell population would be more reasonable the moment that CD3^low^CD20^+^ B cells are observed in freshly analyzed blood samples.

However, it is obvious that human cellular analyses performed *ex vivo*/*in vitro* by different groups reveal interlaboratory variations of results although the populations and techniques employed were similar [22]. Thus, factors including storage time and temperature of samples prior to analysis seem to be crucial for the reproducibility of results [25], [26]. In this regard, a standardized protocol for phenotyping of immune cells by flow cytometry was developed and proposed by the ONE study [26]. In order to facilitate a meaningful comparison between results of different laboratories, human blood samples should be investigated – where possible – within 4–6 hours to prevent storage-induced changes [25], [26]. More precisely, if a delay in blood analysis cannot be avoided, strict monitoring and regulation of temperature are critical to obtain comparable results between different laboratories and different studies, particularly for storage times greater than 24 h [21], [59].

In summary, cellular alterations after storage of human blood samples observed in the present study provide additional insights into the limitations of laboratory *ex vivo*/*in vitro* analyses. This study identified a phenomenon of CD3 acquisition by B cells from T cells, which seems to be a result of non-physiological storage of lymphocytes. It might be possible that additional cell surface molecules (other than CD3 or CD4) are also transferred. Most studies characterizing surface molecule expression on immune cells are performed in an *ex vivo*/*in vitro* setting and many studies present alterations and exchange of cell surface molecules along with clinical diseases without specifying storage conditions of blood samples before experimental analysis. To allow an interpretation of laboratory results, to verify the impact of findings according to immunological diseases and to ensure reproducibility of experimental *ex vivo* findings, we strongly recommend to use standards for flow cytometry analysis (such as postulated by Streitz et al. [26]) or at least to clearly elaborate on the storing conditions of human blood samples in the study design.

## Supporting Information

Figure S1 **Sorting of CD3-expressing CD20^+^ (CD3^low^CD20^+^) lymphocytes after overnight (oN) storage of whole blood samples at 4°C (** ***upper panel*** **) and post-sort analysis of CD3^low^CD20^+^ cells (** ***lower panel*** **).** Doublet/aggregate discrimination was applied by SSC-W vs. SSC-H and FSC-A vs. FSC-W dot plots. Data shown are representative of two experiments performed.
(TIF)
