# Supplementary figures and images for: CD3-Positive B Cells: A Storage-Dependent Phenomenon
Source: PLoS One. 2014 Oct 16;9(10):e110138. doi: 10.1371/journal.pone.0110138 (PMC4199681; doi:10.1371/journal.pone.0110138)

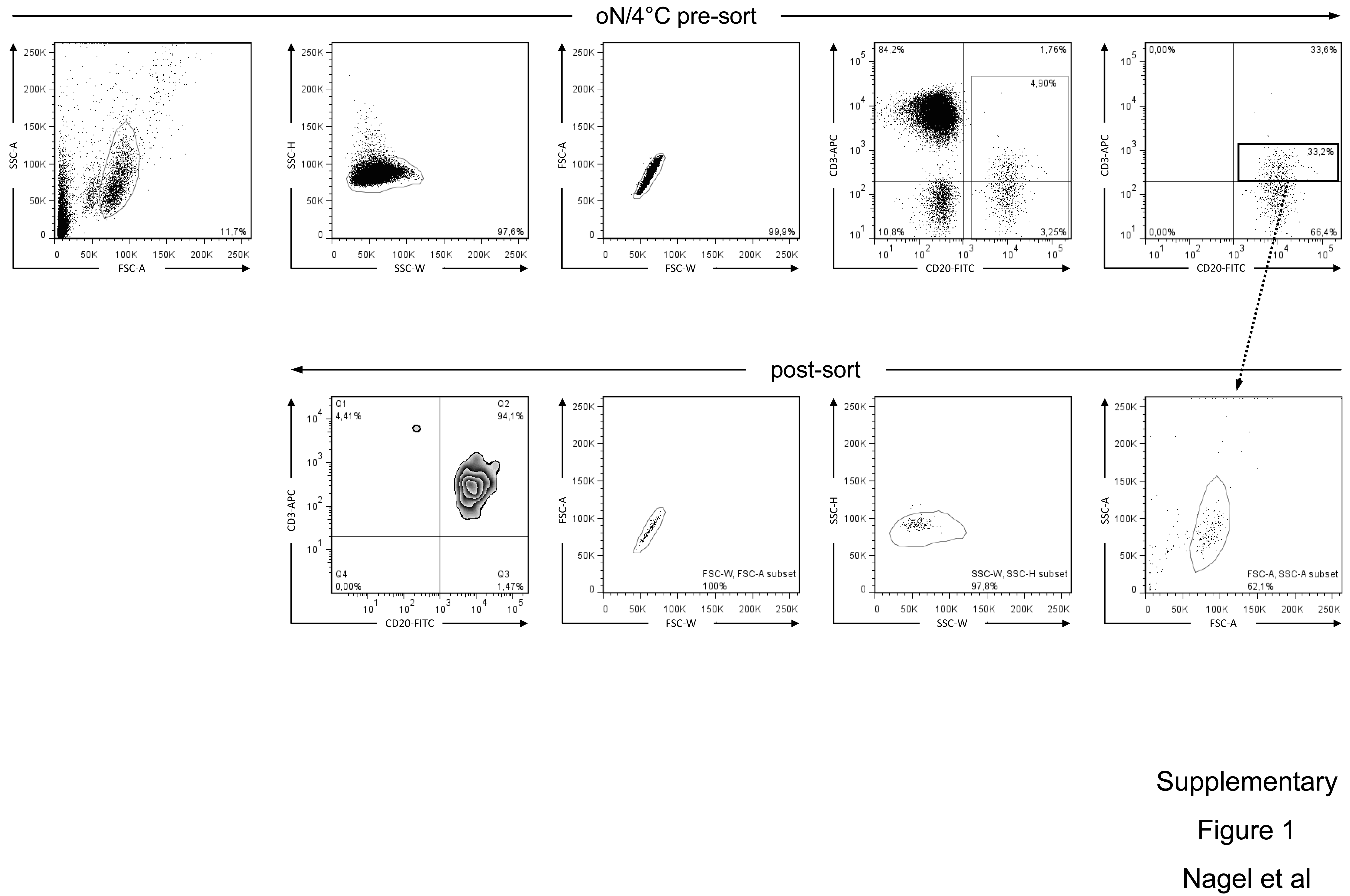

Supplement: Figure S1 — Sorting of CD3-expressing CD20+ (CD3lowCD20+) lymphocytes after overnight (oN) storage of whole blood samples at 4°C ( upper panel ) and post-sort analysis of CD3lowCD20+ cells ( lower panel ). Doublet/aggregate discrimination was applied by SSC-W vs. SSC-H and FSC-A vs. FSC-W dot plots. Data shown are representative of two experiments performed. (TIF) [file pone.0110138.s001.tif]
